# Intronic Binding Sites for hnRNP A/B and hnRNP F/H Proteins Stimulate Pre-mRNA Splicing

**DOI:** 10.1371/journal.pbio.0040021

**Published:** 2006-01-10

**Authors:** Rebeca Martinez-Contreras, Jean-François Fisette, Faiz-ul Hassan Nasim, Richard Madden, Mélanie Cordeau, Benoit Chabot

**Affiliations:** **1** RNA/RNP Group, Département de microbiologie et d'infectiologie, Faculté de médecine et des sciences de la santé, Université de Sherbrooke, Sherbrooke, Québec, Canada; **2** Centre de genomique fonctionnelle de Sherbrooke, Faculté de médecine et des sciences de la santé, Université de Sherbrooke, Sherbrooke, Québec, Canada; University of WisconsinUnited States of America

## Abstract

hnRNP A/B proteins modulate the alternative splicing of several mammalian and viral pre-mRNAs, and are typically viewed as proteins that enforce the activity of splicing silencers. Here we show that intronic hnRNP A/B–binding sites (ABS) can stimulate the in vitro splicing of pre-mRNAs containing artificially enlarged introns. Stimulation of in vitro splicing could also be obtained by providing intronic ABS in *trans* through the use of antisense oligonucleotides containing a non-hybridizing ABS-carrying tail. ABS-tailed oligonucleotides also improved the in vivo inclusion of an alternative exon flanked by an enlarged intron. Notably, binding sites for hnRNP F/H proteins (FBS) replicate the activity of ABS by improving the splicing of an enlarged intron and by modulating 5′ splice-site selection. One hypothesis formulated to explain these effects is that bound hnRNP proteins self-interact to bring in closer proximity the external pair of splice sites. Consistent with this model, positioning FBS or ABS at both ends of an intron was required to stimulate splicing of some pre-mRNAs. In addition, a computational analysis of the configuration of putative FBS and ABS located at the ends of introns supports the view that these motifs have evolved to support cooperative interactions. Our results document a positive role for the hnRNP A/B and hnRNP F/H proteins in generic splicing, and suggest that these proteins may modulate the conformation of mammalian pre-mRNAs.

## Abstract 2

Typically viewed as enforcing splicing silencers, hnRNP A/B proteins may facilitate splicing by modulating the conformation of mammalian pre-mRNAs.

## Introduction

Exons represent approximately 1% of the human genome and range in size from 1 to 1,000 nucleotides (nt), with a mean size for internal exons of 145 nt [1]. In contrast, introns constitute 24% of our genome, with sizes ranging from 60 to more than 200,000 nt. The mean size of human introns is more than 3,300 nt, and nearly 20% of human introns are larger than 5 kb [1]. While the efficient and accurate removal of introns is crucial for the production of functional mRNAs, it remains unclear as to how an intron is defined when splicing junctions are separated by thousands of nucleotides. Although intron size can influence alternative splicing in mammals [2], the mechanisms that enforce the removal of large mammalian introns have not been investigated—partly because introns larger than 1 kb are not spliced efficiently in vitro.

Some of the decisions associated with the removal of large introns are likely to be similar to the choices made by the splicing machinery when selecting alternative splice sites. Choosing the appropriate pair of splice sites in alternative splicing units requires the contribution of many types of elements that are recognized by different classes of proteins, including serine/arginine–residue proteins (SR) and hnRNP proteins. hnRNP A1 was the first protein of its class to be attributed a function in splice-site selection based on its ability to antagonize the activity of the SR protein ASF/SF2 in an in vitro 5′ splice-site selection assay [3]. hnRNP A/B proteins have now been documented to modulate the alternative splicing of many mammalian and viral pre-mRNAs (for a review, see [4]). In one case, an exonic binding site for hnRNP A1 prevents the interaction of positively-acting SR proteins by a process that apparently involves the nucleation of several A1 molecules [5]. Our previous work on the alternative splicing of the hnRNP A1 pre-mRNA led to a different model to explain the activity of hnRNP A/B proteins bound to intronic sites. In this case, bound hnRNP A/B proteins would self-interact to loop out and repress internal splice sites [4,6,7]. This postulated change in pre-mRNA conformation would bring in closer proximity the external pair of exons, an event that may also enforce intron definition. Notably, putative binding sites for hnRNP A/B are more abundant in introns than in exons (1.2 sites and 0.35 sites per 1,000 nt, respectively), and are found more frequently near splice junctions [6].

A similar bias in the distribution of GGG motifs has been observed in mammalian introns [8–16]. GGG motifs located downstream from a 5′ splice site facilitate intron definition [13,17–19], and their insertion near the 5′ splice site of a *Fugu* intron stimulates splicing in a mammalian cell line [15]. Because many binding sites for hnRNP A/B and F/H proteins contain G triples [20–23], hnRNP A/B and hnRNP F/H proteins bound near the ends of an intron may be responsible for the activity of GGG motifs and, hence, may play a generic role in intron definition.

We examined the role of hnRNP A/B and hnRNP F/H proteins in intron removal by making use of the observation that enlarged introns are spliced poorly in vitro. We show that *cis*- and *trans*-acting binding sites for hnRNP A/B and hnRNP F/H proteins (ABS and FBS, respectively) stimulate the splicing of such introns. For some pre-mRNAs, the splicing-enhancing activity of ABS or FBS requires that these sites be positioned at both ends of the intron, consistent with the view that hnRNP A/B and F/H proteins act cooperatively to change pre-mRNA conformation. Overall, our results suggest that hnRNP A/B and hnRNP F/H proteins may stimulate splicing by facilitating intron definition.

## Results

### Binding Sites for hnRNP A/B Proteins Stimulate the In Vitro Splicing of Enlarged Introns

**Figure 1:**
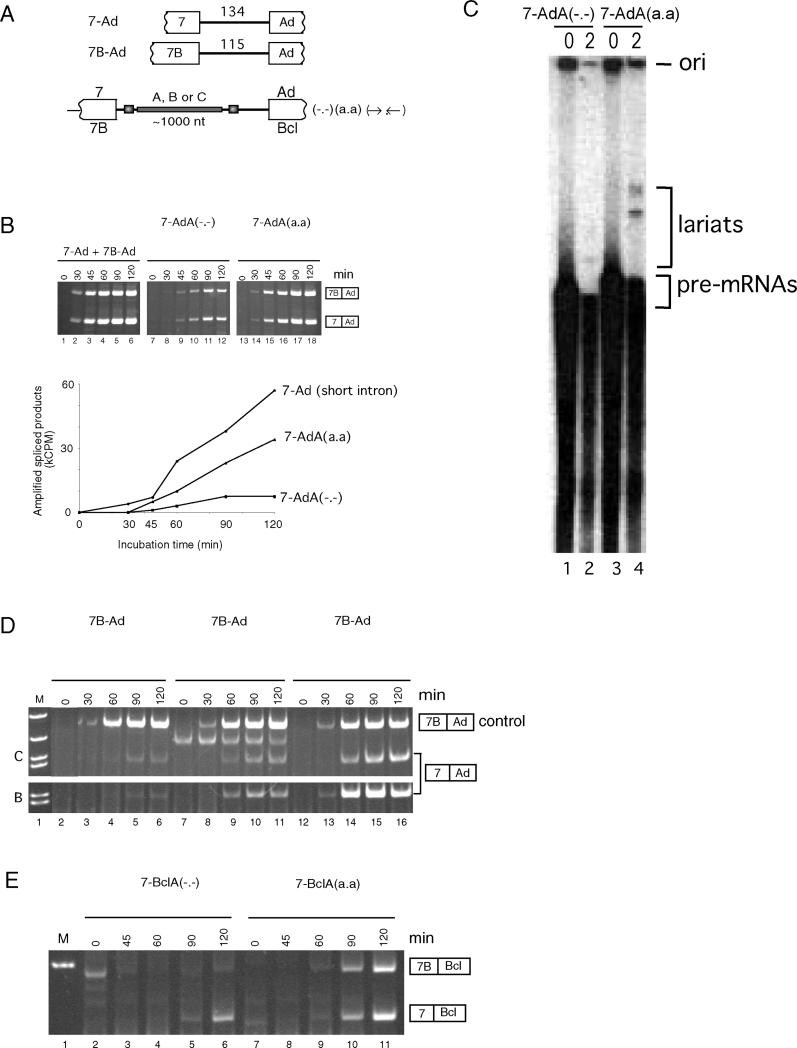
Binding Sites for hnRNP A1/A2 Stimulate the In Vitro Removal of Enlarged Introns (A) The model pre-mRNAs contain portions of exons 7 or 7B of the hnRNP A1 gene paired with the adenovirus L2 exon. The size of the small introns in 7-Ad and 7B-Ad pre-mRNAs is indicated in nt. The size of lambda inserts A, B, and C are, respectively, 1015, 943, and 1038 nt. The lambda inserts do not contain the sequences U AGGG^U^/_A_ or U AGAG^U^/_A_, which correspond to high-affinity binding sites for hnRNP A/B proteins [20,43]. The larger intron substrates contain either exon 7 or exon 7B as first exon, and either the adenovirus L2 or the Bcl-x exon 3 as second exon. When no other elements are inserted, the pre-mRNAs correspond to the (−.−) version. The (a.a) versions contain ABS inserted 26 nt downstream of the 5′ splice site and 88 nt upstream of the 3′ splice site. The (→.←) versions contain inverted repeats at the same positions as ABS. (B) The 7-Ad and 7B-Ad pre-mRNAs were co-incubated for the times indicated (in minutes) in a HeLa nuclear extract (lanes 1–6). Additional mixtures were prepared with pre-mRNAs carrying lambda insert A lacking or containing ABS (lanes 7–12 and 13–18, respectively). The concentration of each pre-mRNA was 80 pM. Following RNA extraction, the mRNA products from mixtures were amplified by RT-PCR using a common set of primers (reverse primer complementary to the adenovirus exonic sequence and forward primer corresponding to plasmid sequence upstream of exon 7 or exon 7B sequences). The graph displays the abundance of amplified splicing product at different times for 7-Ad and the different 7-AdA pre-mRNAs. The RT-PCR assay shown here and in other figures was performed in conditions that displayed a linear relationship between the amounts of input RNA and amplified products over a large range of input RNA concentrations (from 10-fold less to at least 6-fold more than the amounts used in the assays [data not shown]). (C) Splicing reactions were set using ^32^P-labeled pre-mRNAs and incubated for 0 or 2 h in HeLa nuclear extracts. Total RNA was extracted, and the splicing products were fractionated on a 5% acrylamide/8 M urea gel. The position of the lariat products is indicated. (D) Each of the 7-Ad pre-mRNAs carrying lambda inserts B or C (7-AdB or 7-AdC; 80 pM) was co-incubated with the small-intron 7B-Ad pre-mRNA (8 pM). Versions lacking (−.−) or containing ABS (a.a), as well as carrying inverted repeats (→.←), were used. Following incubation for different times, spliced products were amplified by RT-PCR using a common set of primers. The co-incubated small-intron control is only shown for the 7-AdC pre-mRNA mixture. M indicates molecular-weight markers. (E) Large-intron pre-mRNAs 7-BclA and 7B-BclA (80 pM each) lacking (−.−) or containing ABS (a.a) were co-incubated for the indicated times in a HeLa extract. RT-PCR was performed as described in (B) except that a Bcl-x reverse primer was used. The band amplified at *t* = 0 (lane 2) is artifactual and does not co-migrate with the 7B/Bcl splicing product. M indicates molecular-weight markers.

To monitor splicing activity, we first used model pre-mRNAs with small introns that are spliced efficiently in vitro (7-Ad and 7B-Ad; Figure 1A) [7]. As determined by the RT-PCR amplification of the resulting mRNA products, a time-course co-incubation of these two pre-mRNAs in a HeLa nuclear extract indicates that they are spliced with similar efficiencies (Figure 1B, lanes 1–6). Insertion of a 1,015-nt lambda fragment in the intron of each model substrate generated the 7-AdA(−.−) and 7B-AdA(−.−) derivatives. These larger pre-mRNAs were spliced less efficiently than the small-intron versions (Figure 1B, lanes 7–12 and accompanying graph). A splicing efficiency inferior to 1% was estimated by visualizing labeled lariat molecules in a conventional splicing gel (Figure 1C, lane 2). Different lambda inserts of similar lengths had a similar effect on splicing, as seen when the 7-AdB(−.−) and 7-AdC(−.−) pre-mRNAs were co-incubated in splicing mixtures with the small-intron 7B-Ad pre-mRNA (Figure 1D, lanes 2–6). Thus, increasing intron size with a variety of lambda inserts reduced in vitro splicing efficiency.

To determine whether hnRNP A/B proteins could stimulate the splicing of these enlarged introns, an ABS corresponding to the CE1a element in the mouse hnRNP A1 pre-mRNA [6,24] was inserted at two positions in the enlarged introns: one ABS was inserted 26 nt downstream from the 5′ splice junction, while the second ABS was inserted 88 nt upstream from the 3′ splice junction (Figure 1A). The presence of two ABS in the 7-AdA(a.a) and 7B-AdA(a.a) pre-mRNAs stimulated splicing approximately 4-fold (Figure 1B, lanes 13–18 and accompanying graph). Likewise, when lariat splicing products were detected in a conventional splicing gel using ^32^P-labeled transcripts, the presence of ABS stimulated in vitro splicing 3-fold (Figure 1C, lane 4). Splicing stimulation was also observed when ABS were inserted in the 7-AdB and 7-AdC pre-mRNAs (Figure 1D, lanes 7–11). Stimulation was estimated to be in the order of 3- to 5-fold, based on amplification reactions performed in the presence of ^32^P-dCTP (data not shown). Replacing ABS with 20-nt inverted repeats also stimulated large-intron splicing (Figure 1D, lanes 12–16), suggesting that looping out intron sequences can improve the in vitro splicing efficiency of enlarged introns. We also tested the impact of ABS on the splicing of large-intron pre-mRNAs carrying the 3′ splice site of Bcl-x exon 3 (7-BclA and 7B-BclA). The presence of ABS also stimulated the production of amplicons corresponding to mRNA products (Figure 1E; compare the intensities of the 7/Bcl and 7B/Bcl products in lanes 2–6 with those in lanes 7–11).

**Figure 2:**
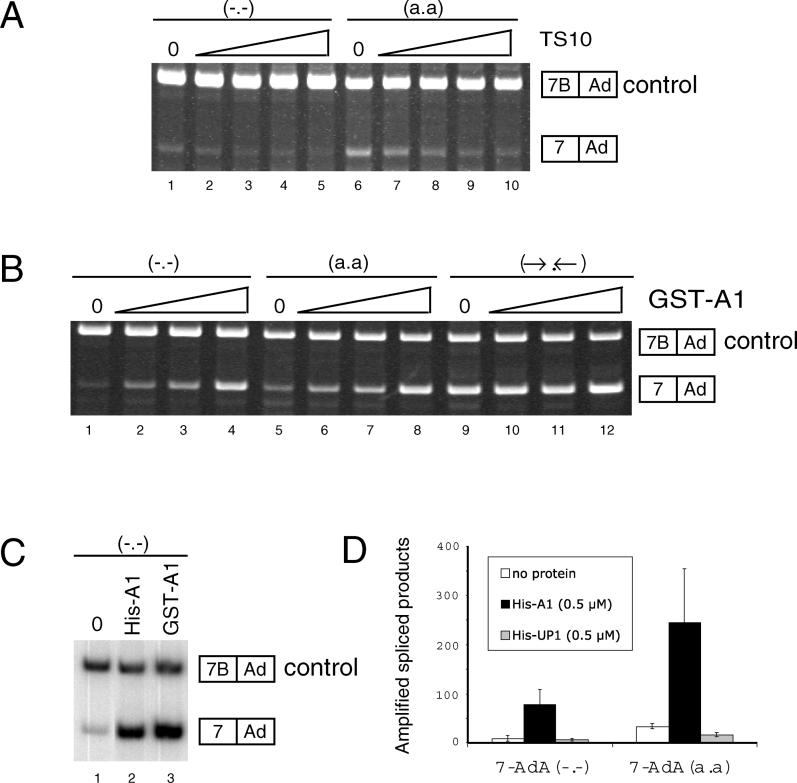
The hnRNP A1 Protein Stimulates Large-Intron Splicing (A) Sequestering hnRNP A/B proteins affects large-intron splicing. The large-intron substrates 7-AdB lacking ABS (−.−) or containing ABS (a.a) were co-incubated with the small-intron 7B-Ad pre-mRNA (80 pM for the 7-AdB substrates and 16 pM for the control 7B-Ad pre-mRNA) in a HeLa extract for 90 min in the presence of increasing amounts of the telomeric oligonucleotide TS10 (0, 80, 160, 320, and 640 nM, respectively). (B) Splicing mixtures were incubated with increasing amounts of recombinant GST-A1 protein (0, 0.8, 1.6, and 3.2 μM). The 7-AdB pre-mRNA carrying inverted repeats (→.←) was also used. (C) Splicing mixtures containing the 7-AdB and the small-intron 7B-Ad were supplemented with His-A1 and GST-A1 (0.5 μM each). (D) The histogram depicts a compilation of three independent experiments performed with the indicated concentrations of recombinant proteins. In each case, individual values obtained for the splicing of 7-AdA (−.−) or 7-AdA (a.a) pre-mRNAs were normalized with the splicing efficiency of the small intron 7B-Ad pre-mRNA. Error bars indicate standard deviations.

To ensure equivalent recovery and loading of the various samples in future experiments, we relied on systematically co-incubating each test pre-mRNA with a control small-intron pre-mRNA in splicing mixtures. mRNA products derived from both substrates could then be amplified simultaneously by RT-PCR using the same primer set. In these conditions, we confirmed that the stimulation offered by ABS was observed at different ratios of test and control pre-mRNAs (Figure S1). To confirm the participation of hnRNP A/B proteins in splicing stimulation, we added to a HeLa nuclear extract increasing amounts of a DNA oligonucleotide (TS10) carrying high-affinity binding sites for A1 and A2 (apparent Kd less than 5 nM, data not shown and see [25,26]). We have shown previously that an excess of TS10 abrogates the activity of ABS in alternative splicing [6]. An excess of TS10 similarly reduced the splicing efficiency of the 7-AdB(a.a) pre-mRNA, without affecting the amplification of splicing products derived from the 7B-Ad small-intron pre-mRNA (Figure 2A, lanes 6–10).

We also tested the impact of increasing the level of hnRNP A1 in the extract by using recombinant A1 protein. GST-A1 stimulated the splicing efficiency of 7-AdB(a.a) (Figure 2B, lanes 5–8). In contrast, splicing of the control small-intron 7B-Ad pre-mRNA was not affected by the addition of GST-A1. GST-A1 also stimulated splicing of the 7-AdB(−.−) pre-mRNA in a dose-dependent manner (Figure 2B, lanes 1–4). The reason for this stimulation is unclear. It is possible that weaker ABS in the large intron of the 7-AdB pre-mRNA are activated when the concentration of hnRNP A1 is increased. In contrast, splicing of the large-intron pre-mRNA carrying inverted repeats was not further stimulated by the addition of GST-A1 (lanes 9–12). A His-tagged version of A1 (His-A1) was as active as GST-A1 at stimulating large-intron splicing (Figure 2C and 2D). In contrast, the addition of His-tagged UP1 (His-UP1), a shortened version of A1 lacking the C-terminal glycine-rich domain, did not stimulate splicing of the large-intron pre-mRNAs (Figure 2D). Rather, His-UP1 slightly impaired splicing of the ABS-containing 7-AdA(a.a) pre-mRNA, possibly because it antagonized the binding of endogenous hnRNP A/B proteins.

**Figure 3:**
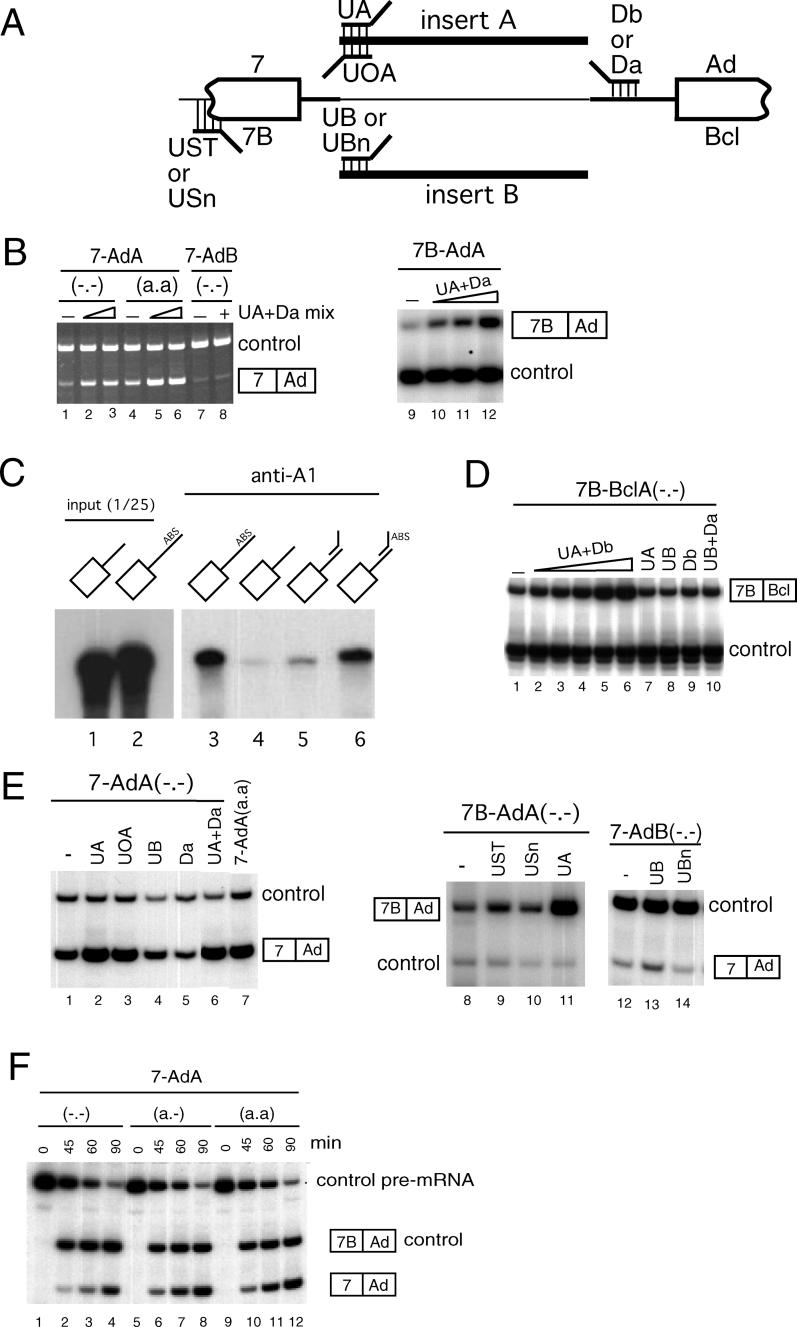
Antisense Oligonucleotides Carrying ABS Stimulate the Splicing of Large Introns (A) Schematic representation of model large-intron pre-mRNAs and the position and structure of the RNA oligonucleotides. Oligonucleotides UA and UB form a duplex with sequences located 46–65 and 46–64 nt, respectively, downstream from the 5′ splice site. Oligonucleotides Db and Da, respectively, hybridize 123–142 nt and 68–87 nt upstream of the 3′ splice junction. (B) The 7-AdA pre-mRNA lacking ABS (−.−) or containing ABS (a.a) was incubated in a HeLa extract in the absence (lanes 1 and 4) or in the presence (lanes 2 and 3, and lanes 5 and 6) of UA and Da oligonucleotides (16 and 40 nM of each oligonucleotide). The 7-AdB pre-mRNA (−.−) and the 7B-AdA pre-mRNAs were also incubated in the presence of UA and Da (40 nM in lane 8, and 0.08, 0.8, and 8 nM in lanes 10–12, respectively). As internal control for splicing, a smaller quantity of the small-intron 7B-Ad pre-mRNA (lanes 1–8) or the small-intron 7-Ad pre-mRNA (lanes 9–12) was co-incubated with the test substrates. Incubation in HeLa extracts was for 60 min. (C) Labeled transcripts corresponding to the first 196 of the 7-AdA(−.−) pre-mRNA (lanes 1, 4, 5, and 6) or the first 237 nt of the ABS-containing 7-AdA(a.a) pre-mRNA (lanes 2 and 3) were incubated in a HeLa nuclear extract in the presence of 100 pM of the ABS-lacking UAn oligonucleotide (lane 5) or the ABS-containing UA (lane 6). Mixtures were immunoprecipitated with an anti-hnRNP A1 antibody and resolved in a denaturing 5% polyacrylamide gel. The initial input for each transcript representing 1/25th of the total amount is shown in lanes 1 and 2. (D) The 7B-BclA was co-incubated with 100-fold less of the small-intron 7-Ad control pre-mRNA and increasing amounts of the UA and Db oligonucleotide mixture (0, 0.08, 0.8, 8, 80, and 160 nM) or with 160 nM of individual or mixtures of various oligonucleotides. (E) The 7-AdA pre-mRNA was co-incubated with the small-intron 7B-Ad pre-mRNA in a HeLa nuclear extract for 90 min at 30 °C. Each oligonucleotide was used at a concentration of 160 nM. The 7-AdA(a.a) pre-mRNA containing *cis*-acting ABS elements was used as a control (lane 7). In lanes 8–11, the 7B-AdA pre-mRNA was co-incubated with the small-intron 7-Ad pre-mRNA and 80 nM of oligonucleotides. In lanes 12–14, the 7-AdB pre-mRNA was co-incubated with the 7B-Ad control pre-mRNA and either the UB or UBn (40 nM each). UBn carries a non-ABS tail. Incubation was for 60 min in a HeLa extract. (F) The control 7B-Ad pre-mRNA was co-incubated with 7-AdA pre-mRNA containing either no ABS (−.−), only the upstream ABS (a.−), or two ABS (a.a). Incubation in HeLa extracts was for 0, 45, 60, and 90 min. RT-PCR assays performed with a single pair of primer allows amplification of the unspliced control pre-mRNA, as well as mRNA products derived from both the control and the 7-AdA derivatives.

We have recently used antisense oligonucleotides carrying a non-hybridizing ABS-containing tail to alter splicing decisions in vitro and in vivo [27]. The ABS tail interfered with splicing when the antisense portion of the oligonucleotide was complementary to exonic sequences upstream of 5′ splice sites. To verify whether ABS-tailed oligonucleotides could now act positively by reproducing the effect of *cis*-acting ABS, we added to a HeLa extract a mixture of *trans*-acting ABS-containing oligonucleotides complementary to the ends of the introns in the 7-AdA(−.−) and 7B-AdA(−.−) pre-mRNAs (UA and Da; Figure 3A). The in vitro splicing of these pre-mRNAs was stimulated in both cases (Figure 3B, lanes 1–3 and lanes 9–12, respectively). The UA and Da mixture also stimulated splicing of the 7-AdA(a.a) pre-mRNA (Figure 3B, lanes 4–6). In contrast, the oligonucleotide mixture did not improve splicing of a pre-mRNA carrying the B insert (lanes 7 and 8). In general, concentrations of oligonucleotides varying between 0.08 and 160 nM stimulated splicing (representing a molar excess of 10- to 2,000-fold relative to the pre-mRNA). The level of stimulation ranged from 2- to 8-fold between different experiments (data not shown). Concentrations superior to 160 nM usually promoted a specific reduction in the splicing efficiency of enlarged introns, possibly because of titration of hnRNP A/B proteins by an excess of ABS-containing oligonucleotides (data not shown).

Our results with the ABS-containing oligonucleotide mixture therefore suggest that hnRNP A/B proteins can be recruited at the intended positions in the intron. This conclusion was supported by the results of an immunoprecipitation assay performed in nuclear extracts using an anti-A1 antibody and a portion of the 7-AdA pre-mRNA. The results show that recovery of the RNA is improved by the presence of a *cis*-acting ABS (Figure 3C, compare lanes 3 and 4). Likewise, providing a *trans*-acting ABS as part of the tail of an antisense oligonucleotide stimulates recovery (lane 6), a result not observed when the oligonucleotide carries a non-ABS tail (lane 5). Thus, a *cis*- or *trans*-acting ABS improves the association of hnRNP A1 with the target RNA.

We have recently proposed that the mechanism underlying the activity of hnRNP A/B proteins in alternative splicing involves an interaction between bound A/B proteins such that portions of the pre-mRNA are looped out, therefore changing pre-mRNA structure to favor contacts between the external pair of exons [6]. One prediction from this model is that the presence of ABS at both ends of the enlarged intron should be required to observe stimulation of splicing. We used *trans*-acting ABS-containing oligonucleotides to assess whether providing an ABS at either the upstream or the downstream position could stimulate splicing. First, we present the activity of individual *trans*-acting ABS on a pre-mRNA carrying the 3′ splice site of human Bcl-x exon 3 (7B-BclA). The addition of oligonucleotides UA and Db stimulated 7B/Bcl splicing (Figure 3D, lanes 1–6), a result that reproduced the activity of *cis*-acting ABS (Figure 1E). Stimulation required the presence of both oligonucleotides since UA or Db alone did not stimulate splicing (Figure 3D, lanes 7 and 9).

Providing a non-hybridizing oligonucleotide with an ABS tail was inactive (lane 8), and combining this oligonucleotide with the non-hybridizing Da oligonucleotide also did not provide stimulation (lane 10). Thus, stimulation of 7B-BclA pre-mRNA splicing required a combination of upstream and downstream *trans*-acting ABS, suggesting cooperative interactions between these sites, in accord with the looping-out model. Second, we tested the impact of individual oligonucleotides on pre-mRNAs carrying the adenovirus 3′ splice site (7-AdA). Notably, the upstream UA oligonucleotide alone stimulated 7-AdA splicing almost as efficiently as the UA and Da mixture (Figure 3E, lanes 2 and 6). The UA oligonucleotide alone also stimulated 7B-AdA splicing (lane 11). In contrast, the downstream Da oligonucleotide alone did not stimulate splicing (lane 5). Providing the ABS as a 3′ rather than a 5′ extension was also stimulatory (UOA; Figure 3D, lane 3), and no activity was provided by an oligonucleotide carrying a non-ABS extension (UBn; lane 14), or an ABS-containing oligonucleotide complementary to the first exon (UST; lane 9). A single *cis*-acting ABS at the upstream position in the large intron of the 7-AdA also stimulated splicing (7-AdA[a.−]; Figure 3F). *Trans*-acting ABS hybridizing at a distance greater than 250 nt from the 5′ splice site did not significantly enhance splicing (data not shown).

Likewise, placing two *cis*-acting ABS in the middle of an enlarged intron did not stimulate splicing (data not shown). Thus, while positioning an ABS relatively close to the 5′ splice site is apparently important for splicing stimulation, a single *cis*- or *trans*-acting ABS positioned near the 5′ splice site is sufficient for stimulating splicing of the 7-AdA and 7B-AdA pre-mRNAs. This conclusion contrasts with the results obtained with the 7B-BclA pre-mRNA and does not a priori support the looping-out model. Although the reason for this difference remains unclear, hnRNP A1 has been reported to bind to the adenovirus 3′ splice site [28]. Thus, a high-affinity A1-binding site located at this position may collaborate with an ABS near the 5′ splice site to stimulate pre-mRNA splicing. Unfortunately, we could not confirm this hypothesis experimentally because the putative ABS directly overlaps the adenovirus 3′ splice site, and mutating the ABS would inhibit splicing.

### Antisense Oligonucleotides Carrying ABS Stimulate Splicing of an Enlarged Intron In Vivo

**Figure 4:**
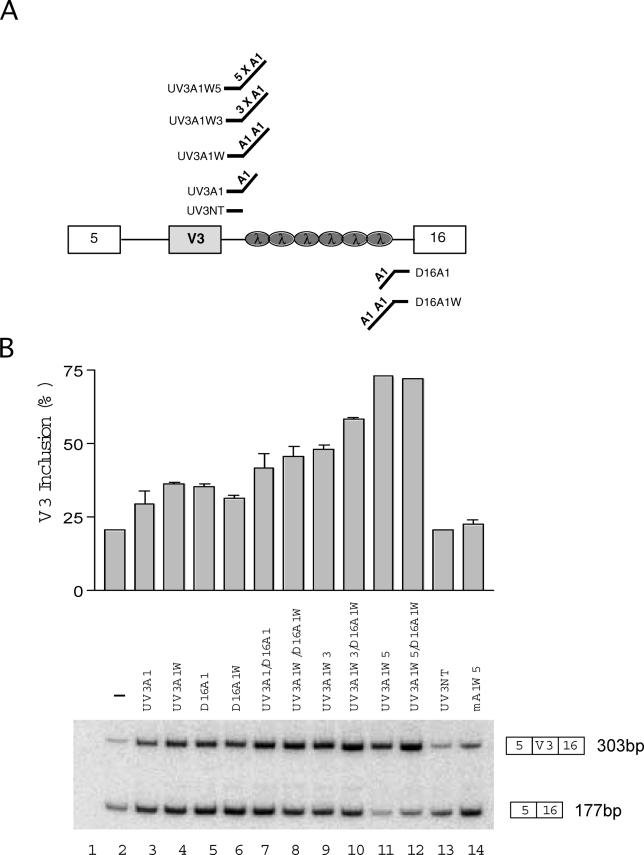
The In Vivo Splicing of a Large Intron Is Improved by ABS-Containing Oligonucleotides (A) Structure of the p44:v3λλλλλλ pre-mRNA. This pre-mRNA contains the alternatively spliced V3 exon as well as constitutive exons 5 and 16 from the *CD44* gene. Six 1-kb lambda DNA repeats (gray circles) were inserted downstream of V3 to increase the length of this intron [2]. The position and structure of the RNA oligonucleotides are depicted. A1 indicates an ABS element. (B) COS-7 cells were transfected with plasmid p44:V3λλλλλλ. Twenty-four hours later, they were treated with different 2′*O*-Me RNA oligonucleotides, and total RNA was extracted after 24 h. A RT-PCR assay was performed in the presence of (^32^P)dCTP to determine the relative levels of both mRNA splicing products. The inclusion frequency of exon V3 expressed as a percentage is shown graphically with mean value and error bars derived from three separate experiments.

To address whether ABS can stimulate intron splicing in vivo, we relied on a model pre-mRNA used previously to demonstrate the negative influence of intron size on exon inclusion [2]. The *CD44* model pre-mRNA contains the V3 alternative exon flanked downstream by an enlarged intron containing six adjacent 977-nt spacer elements derived from lambda DNA (Figure 4A). In this configuration, the enlarged intron promotes exon V3 skipping [2]. We tested the effect of providing *trans*-acting ABS at one or both ends of the enlarged intron. Following transfection of the *CD44* plasmid in COS-7 cells, a second transfection was performed with antisense 2′*O*-Me oligonucleotides carrying an ABS tail. hnRNP A1 binds very well to 2′*O*-Me RNA [27]. Compared to a control oligonucleotide lacking an ABS tail (UV3NT; Figure 4B), oligonucleotides complementary to the 5′ end of the intron and carrying only one ABS stimulated exon V3 inclusion very slightly (from 20% with UV3NT to 29% with UV3A1 with a *p-*value of 0.1 based on three separate experiments). A more important stimulation was obtained when the tailed oligonucleotide carried two, three, or five ABS (UV3A1W, UV3A1W3, and UV3A1W5, respectively, promoting an average of 36%, 48%, and 73% inclusion). A control oligonucleotide with five adjacent ABS but lacking a portion complementary to the *CD44* mini-gene did not improve exon V3 inclusion (mA1W5). Exon inclusion was also stimulated by tailed oligonucleotides complementary to the 3′ portion of the intron (D16A1 and D16A1W).

Providing oligonucleotides as mixtures (UV3A1/D16A1, UV3A1W/D16A1W, UV3A1W3/D16A1W, or UV3A1W5/D16A1W) offered little or no additional stimulation when compared to the effect of providing the upstream oligonucleotide alone. Consistent with our previous study [27], the activity of the ABS-tailed oligonucleotides was compromised when the concentration of hnRNP A1/A2 proteins was reduced through the use of siRNAs targeting the A1/A2 mRNAs (data not shown). Thus, oligonucleotides designed to deliver an ABS at the 5′ or the 3′ end of the intron stimulated splicing of the enlarged intron in vivo. However, this experiment did not reveal an apparent cooperation between terminal ABS (see Discussion).

### FBS Duplicate the In Vitro Modulating Activity of ABS

**Figure 5:**
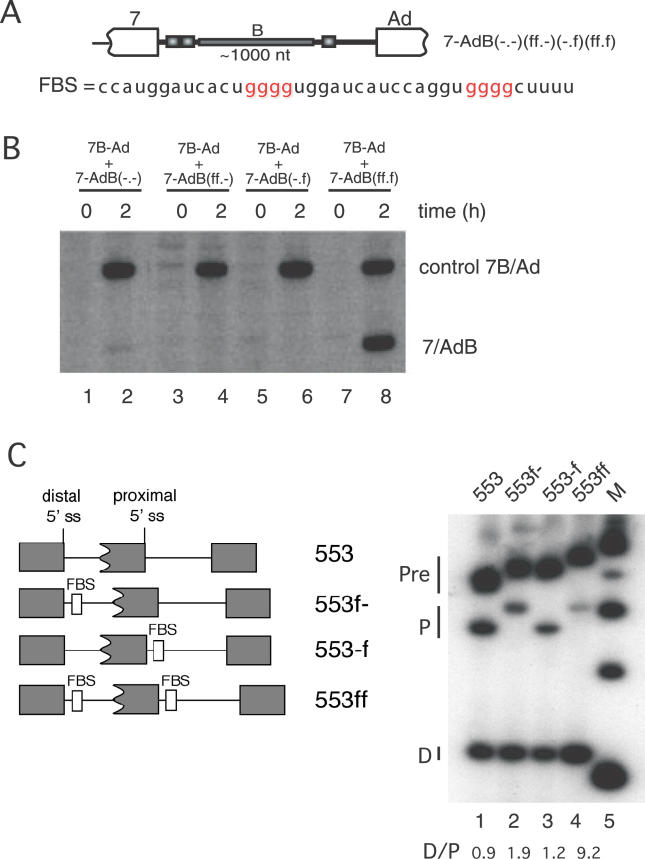
Binding Sites for hnRNP F/H Proteins Stimulate the Splicing of an Enlarged Intron and Modulate 5′ Splice-Site Selection (A) Structure of the pre-mRNA containing the enlarged intron (lambda insert B). The FBS was as described previously [29]. The first 6 nt of the FBS are derived from a NcoI site used for cloning purposes. (B) The control 7B-Ad pre-mRNA (8.7 pM) and the 7-AdB pre-mRNAs (85 pM) containing either no FBS (−.−), two FBS at the upstream position (ff.−), one FBS at the downstream position (−.f), or FBS at both positions (ff.f) were co-incubated for the times indicated (in hours) in a HeLa nuclear extract. A RT-PCR assay was performed to amplify simultaneously spliced mRNAs derived from 7B-Ad or the 7-AdB derivatives. (C) Activity of FBS in 5′ splice-site selection. The structure of the pre-mRNA containing an FBS at one or both positions is shown on the right. The pre-mRNAs were incubated in a HeLa extract for 2 h. A RT-PCR assay was performed to amplify splicing products from splicing mixtures. M indicates molecular-weight markers. The position of the products generated from the use of the distal (D) and proximal (P) 5′ splice sites, as well as from the pre-mRNAs (Pre), is shown. The distal–proximal ratio of products (D/P) is indicated below the lane number.

hnRNP F/H proteins have affinity for G-stretches [23]. Thus, a subset of the GGG motifs found near the ends of mammalian introns may be bound by hnRNP F/H proteins. Moreover, hnRNP F/H proteins contain glycine-rich domains, and similar domains promote an interaction between hnRNP A1 proteins. For these reasons, we tested whether an FBS could stimulate the in vitro splicing of an enlarged intron. We used the hnRNP H-binding site identified in the cystathionine β-synthase gene [29], a site that contains 2 G quadruples (Figure 5A). The insertion of two copies of this FBS at the upstream position in the intron of the 7-AdB pre-mRNA did not stimulate in vitro splicing (Figure 5B, lanes 2 and 4). Likewise, inserting one FBS at the downstream position of the enlarged intron did not improve splicing efficiency (Figure 5B, lane 6). However, when both the upstream and the downstream FBS were present, splicing was strongly stimulated (Figure 5B, lane 8).

To further explore the ability of FBS to mimic ABS, FBS were inserted into a model pre-mRNA to monitor the impact of FBS on 5′ splice-site selection. We used the 553 pre-mRNA which contains the 5′ splice sites of hnRNP A1 exons 7 and 7B joined to the downstream adenovirus 3′ splice site [7]. Inserting one FBS immediately downstream of exon 7 or exon 7B only slightly stimulated distal 5′ splice-site selection (553f- and 553-f; Figure 5C, lanes 1–3). In contrast, the presence of FBS at both positions promoted a strong increase in distal 5′ splice-site usage (553ff; Figure 5C, lane 4), thereby duplicating the impact of ABS in 5′ splice-site selection [7]. Thus, the FBS elements functioned cooperatively in vitro to stimulate distal 5′ splice-site selection and splicing of an enlarged intron.

**Figure 6:**
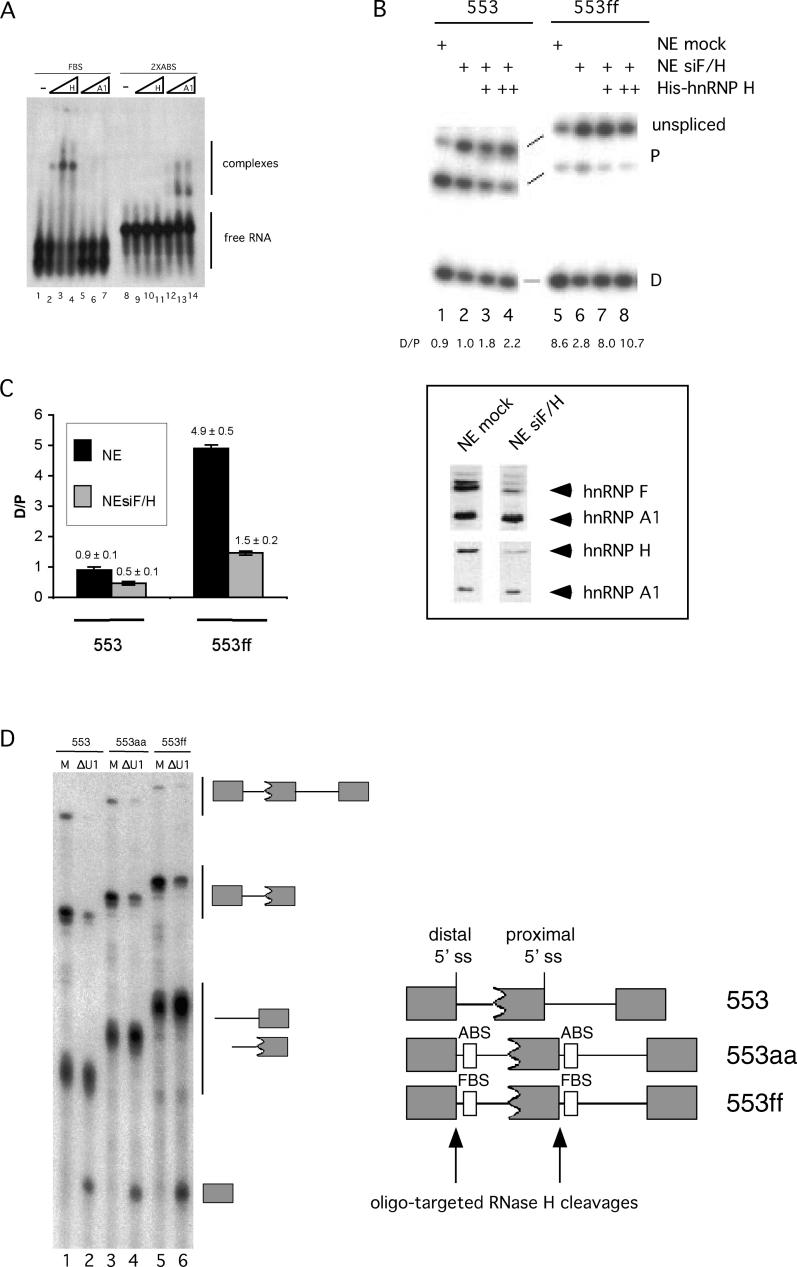
Role of hnRNP F/H Proteins in the Activity of FBS (A) hnRNP H binds FBS specifically. The binding of hnRNP H and hnRNP A1 to FBS and ABS was monitored using a gel-shift assay. The sequence of the FBS RNA corresponds to the sequence shown in Figure 4A, while 2XABS is a 2′*O*-Me oligonucleotide that contains two U AGAGU elements (CCUUU AGAGUAGU AGAGU AGAAUAAG-CCUUGCAUAAAUGG). Binding conditions were as described previously [43] and used 1.25, 2.5, and 3 μM of hnRNP H or A1 proteins. (B) hnRNP F/H are required for the activity of FBS on 5′ splice-site selection. Nuclear extracts were prepared from HeLa cells that were treated with siRNAs against hnRNP F/H [34]. Pre-mRNA substrates lacking or containing FBS were assayed in extracts prepared from mock-treated and siF/H-treated cells. The siF/H extract was also supplemented with recombinant His-tagged hnRNP H protein prepared from baculovirus-infected cells (0.15 μM). The ratio of the products resulting from the use of the distal or proximal 5′ splice site is indicated below the lane number. The right panel shows a Western blot analysis of the content of hnRNP F and H proteins in extracts prepared from mock-treated and siF/H-treated cells. In addition to the anti-F or anti-H antibody, an anti-A1 antibody [45] was co-incubated to reveal A1 and monitor total protein loading. (C) Splicing assays using the 553 and the 553ff pre-mRNAs were performed in triplicate in extracts prepared from mock-treated and siF/H-treated HeLa cells. The ratio of the amplified products corresponding to the proximal and distal 5′ splice-site usage was calculated and plotted in a graph that displays error bars. (D) Oligonucleotide-mediated RNase H protection assays to monitor U1 snRNP occupancy on the competing 5′ splice sites. Pre-mRNAs lacking or containing FBS or ABS were incubated at 0 °C in mock-treated and U1 snRNP-inactivated extracts (ΔU1). Oligonucleotides complementary to the 5′ splice sites were added along with RNase H. The position of the fully protected pre-mRNAs and cleaved molecules is shown.

The contribution of hnRNP F/H proteins in the activity of the FBS element was confirmed in various ways. First, a gel-shift assay indicated that recombinant hnRNP H protein, but not hnRNP A1, bound the FBS element (Figure 6A, lanes 1–7). In contrast, an oligonucleotide carrying two copies of the A1-binding motif used in ABS (U AGAGU) had more affinity for hnRNP A1 than hnRNP H (lanes 8–14). Second, the distal 5′ splice-site promoting activity of FBS was dependent upon hnRNP F/H proteins. This was shown by incubating the 553ff pre-mRNA in a nuclear extract prepared from HeLa cells that had been treated with siRNAs to knockdown hnRNP F/H expression (Figure 6B, right panel). The distal–proximal ratio of products derived from the 553ff pre-mRNA was decreased in such an extract, whereas this ratio was minimally affected with the 553 pre-mRNA (Figure 6B, lanes 5 and 6, and lanes 1 and 2, respectively; see also Figure 6C). Moreover, while the addition of recombinant hnRNP H protein only had a modest effect on 553 pre-mRNA splicing, the distal–proximal ratio of products derived from 553ff pre-mRNA splicing was improved when hnRNP H was added to the siF/H extract (Figure 6B, lanes 3–4 and 7–8, respectively). These results suggest that the activity of the FBS in the HeLa extract requires at least the hnRNP H protein.

We further asked whether the strong shift in 5′ splice-site selection mediated by the pair of FBS was associated with a corresponding change in the binding of U1 snRNP to 5′ splice sites. To assess U1 snRNP binding, we performed an oligonucleotide-mediated RNase H protection assay using a mixture of oligonucleotides complementary to the 5′ splice sites of exon 7 and exon 7B [24]. The protection profile obtained at 0 °C with the control 553 pre-mRNA indicates two populations of U1-bound pre-mRNAs; one bound only to the distal 5′ splice site, and a less abundant population (15%) to which U1 is bound to both the distal and the proximal 5′ splice sites (Figure 6D, lane 1). For pre-mRNAs carrying ABS or FBS, the percentage of transcripts bound by U1 at both the distal and the proximal 5′ splice sites was reduced slightly (11% and 4%, respectively; Figure 6D, lanes 3 and 5). The protection observed in all cases was largely U1 snRNP-dependent (lanes 2, 4, and 6). Our results therefore indicate that the robust improvement in distal 5′ splice-site usage mediated by FBS and ABS was not accompanied by equivalent changes in U1 snRNP binding to the competing 5′ splice sites.

## Discussion

### Stimulation of Splicing by Intronic Binding Sites for hnRNP A/B and hnRNP F/H Proteins

The initial demonstration that hnRNP A1 antagonized the activity of SR proteins in splice-site selection assays was followed by many reports implicating the hnRNP A/B proteins in the activity of exonic silencer elements. Understandably, these findings led hnRNP A/B proteins to be regarded mostly as negative regulators of splicing. The results presented here suggest that the binding of hnRNP A/B proteins in introns can also play a positive role in the generic splicing reaction. Using model pre-mRNAs harboring artificially enlarged introns that are spliced poorly in HeLa nuclear extracts, we have shown that intronic high-affinity ABS positioned near splice junctions can stimulate in vitro splicing.

On the other hand, hnRNP F/H proteins have been implicated in the activity of both splicing enhancers and silencers. For example, the hnRNP F and H proteins are part of a complex assembling on an intronic enhancer element that promotes the neuro-specific inclusion of the N1 exon in the *src* pre-mRNA [21,30]. hnRNP H also activates an SC35-bound exonic enhancer element in the human immunodeficiency virus [31], but is required for the activity of a silencer element located in a rat β-tropomyosin alternative exon [32]. Binding sites for hnRNP H that overlap 5′ or 3′ splice sites can also repress splicing [16,29,33]. Recently, we uncovered a positive role for hnRNP F/H proteins when bound downstream of the Bcl-x_S_ 5′ splice site [34]. We now add to this list of activities the observation that binding sites for hnRNP F/H located at the ends of an enlarged intron can stimulate in vitro splicing. The ability of FBS to replicate the activity of ABS is not limited to the splicing of enlarged introns since FBS also promoted distal 5′ splice-site utilization. This situation contrasts with a recent report documenting a complex interplay between exonic hnRNP A1-binding sites and an intronic GGGG motif in the inclusion of the brain-specific *GRIN1* CI exon [16]. In this case, hnRNP H binding to a GGGG motif appears to antagonize the silencing activity of exonic A1-binding sites. These results suggest that hnRNP A1 and H may exhibit different roles depending on the precise arrangement of their respective binding sites relative to a 5′ splice site.

### hnRNP Proteins and the Looping-Out Model

**Figure 7:**
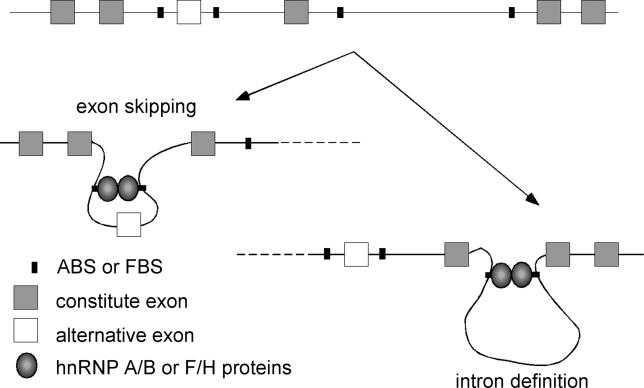
The Looping-Out Model of Action for hnRNP A/B and hnRNP F/H Proteins hnRNP proteins bound to high-affinity binding sites (ABS or FBS) would self-interact to loop out intron sequences and stimulate intron definition. A similar interaction involving ABS or FBS located in distinct introns would loop out an alternative splice site or a cassette exon to favor skipping and commitment between the external pair of splice sites. It remains unknown as to whether heterotypic interactions can occur between hnRNP A/B and hnRNP F/H proteins.

Importantly, upstream and downstream binding sites for hnRNP F/H or hnRNP A/B proteins apparently cooperate in some pre-mRNAs. A pair of ABS was required to stimulate splicing of the 7B-BclA pre-mRNA. A similar requirement was noted for FBS to stimulate the splicing of an enlarged intron. These results are consistent with the view that the mechanism underlying the stimulatory activity of hnRNP A/B and F/H proteins involves an interaction involving terminally bound proteins, such that a portion of the intron is looped out to bring into closer proximity distantly separated exons (Figure 7). The situation was different when we used pre-mRNAs carrying the adenovirus major late 3′ splice site. In this case, an ABS positioned near the 5′ splice site was sufficient for stimulation. Although we cannot rule out alternative explanations, the fact that the adenovirus 3′ splice site is bound by hnRNP A1 [28] may explain why an upstream ABS is sufficient for stimulating splicing of pre-mRNAs carrying this 3′ splice site. Cooperation was also not observed when we targeted the enlarged intron of flanking *CD44* exon V3 in vivo. In this case, we noted that each of the six 977-nt lambda inserts contain a putative ABS (U AGGGU) at position 666. Thus, we propose that an internal ABS may cooperate with a terminal *trans*-acting ABS to stimulate intron removal.

The activity of ABS and FBS that is documented here is relevant to the reported activity of GGG motifs that are found more abundantly near the ends of introns [15]. Such motifs have been associated with an ability to modulate 5′ splice-site selection and facilitate intron splicing [13,15,17–19]. All binding sites for hnRNP F/H proteins characterized so far contain GGG, but it remains unclear as to whether all GGG motifs are bound by hnRNP F/H proteins, since U1 snRNP and SF1 have also been proposed to bind to GGG motifs [18,35]. We tested hnRNP A1, F, and H binding to the two G-rich elements that improve splicing of a *Fugu* intron in mammalian cells [15]. As judged by gel-shift analysis, the two GGG motifs in G1 are not bound by F, H, or A1, whereas the G2 element, which contains a G triple and a GGGG, is bound strongly by hnRNP H but not by either hnRNP F or hnRNP A1 (Figure S2). Although the winner high-affinity site for A1 contains GGG [20], the A1-binding site that we used (U AGAGU) lacks a G triplet and is not bound by hnRNP H. Thus, many GGG motifs found near the ends of introns may be binding sites for hnRNP A/B and F/H proteins, and they may contribute to intron definition.

According to the simplest version of the looping-out model, hnRNP A/B or F/H proteins bound near the ends of introns would interact with one another to loop out most of the intron. It is possible, however, that proteins bound to terminal ABS or FBS also interact with proteins bound to internal sites to loop out portions of the intron. In this case, terminal ABS may ensure a maximal effect on intron definition. To examine the generality of the potential importance of terminal ABS and FBS, we performed a computational analysis for the presence of intronic ABS and FBS at the ends of 156,525 human introns of sizes ≥330 nt (Table 1). Consistent with previous studies, we find that introns carrying one or more GGG motifs near the 5′ splice site (from positions +11 to +150, relative to the 5′ splice junction) or near the 3′ splice site (from positions −41 to −180, relative to the 3′ splice junction) are significantly overrepresented when compared to the values obtained if the sequence in these regions is randomly shuffled (Table 1, *sh*).

**Table 1:**
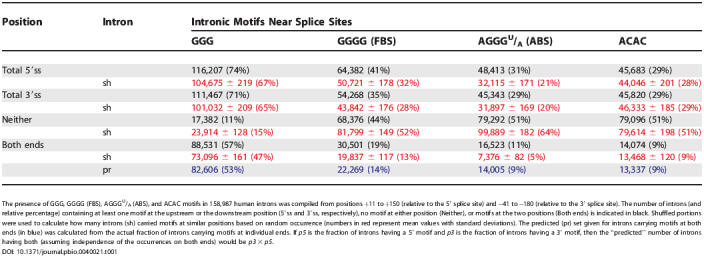
Overrepresentation of Human Introns Carrying FBS or ABS at Both Ends

Next, we asked whether there was a similar enrichment for introns carrying GGG motifs at both ends of the same intron. Interestingly, 8,8531 introns carried at least one GGG at both ends, a number significantly above the predicted number (82,606) based on the actual number of introns with GGG motifs at either the 5′ or the 3′ end (Table 1, *pr*). To discriminate between FBS and ABS, we repeated the analysis using the GGGG motif as an FBS, and the AGGG^U^/_A_ motif as an ABS. Notably, 30,501 introns carried at least one GGGG motif at both extremities, representing an excess of 8,232 introns relative to the number predicted if occurrences at each end were unlinked. A slightly less important enrichment was observed when the analysis was performed with the ABS motif. In this case, 16,523 introns contained at least one ABS motif at both ends, representing an excess of more than 2,518 introns. As a control, we carried out the analysis with the sequence ACAC. Introns carrying this motif at one or both ends were not significantly overrepresented (Table 1). Thus, our results are consistent with the view that ABS and FBS present at both ends of introns cooperate to function in splicing. We noted that there was no bias in the configuration of these motifs according to intron length (data not shown), suggesting that cooperative interactions involving terminal ABS or FBS may occur in a large fraction of introns irrespective of their sizes.

The looping-out model is also the simplest way to explain the behavior of ABS and FBS in 5′ splice-site selection. A pair of FBS was considerably more active than individual FBS at shifting splicing towards the distal 5′ splice site (Figure 5C). Such cooperation was not observed for ABS since distal 5′ splice-site usage was significantly stimulated by positioning an ABS downtream of either the proximal or the distal 5′ splice site [7]. Because this pre-mRNA contains the same adenovirus 3′ splice site as the one used in large-intron substrates, a contribution of the ABS at the 3′ splice site may also contribute to explain this apparent lack of cooperation. Thus, appropriately positioned ABS or FBS can promote alternative 5′ splice-site usage and, possibly, exon skipping (Figure 7). Consistent with this view, we have shown that deleting the intronic ABS flanking constitutive exon 7 and/or alternative exon 7B in the hnRNP A1 pre-mRNA promotes exon 7B inclusion [6].

Additional experimental evidence indirectly supports the looping-out model. A1 proteins self-interact [36], and A1 molecules bound to one ABS can simultaneously interact with another ABS [6]. As these activities require the glycine-rich domain of hnRNP A1, it is notable that hnRNP F/H proteins also contain glycine-rich domains, and that an interaction between hnRNP F and H has been described [30]. The crystal structure of UP1 bound to high-affinity DNA-binding sites is also consistent with the looping model [37]. UP1 contains the two RNA-recognition motifs (RRMs) but lacks the C-terminal glycine-rich domain of A1. In the co-crystal, UP1 exists as a dimer, the RRM1 of each UP1 molecule being bound to sites located on two distinct oligonucleotides. Although UP1 lacks the activity of A1 in splicing and does not self-interact in biochemical and two-hybrid assays [36], the very high concentration of proteins used to promote crystal formation may have forced UP1 dimerization. Thus, we envision that A1 proteins individually bound to distinct high-affinity sites may interact through their glycine-rich domains. This A1–A1 interaction would bring into close proximity the two RNA regions, and the RRM2 domains of each A1 molecule may subsequently cross-interact with sequences flanking the other ABS to stabilize the complex. We speculate that the RRM2 domain of A1 may engage in this type of interaction because the RRM1 is sufficient for specific binding to one high-affinity site [38]. Finally, we have shown that providing FBS or ABS near the ends of an enlarged intron is functionally equivalent to having inverted repeats at these positions. Duplex-forming elements are present in many yeast introns to facilitate commitment between pairs of splice sites [39–42]. It is intriguing to consider that interactions between hnRNP proteins, rather than duplex formation, may have been selected to help in defining mammalian introns. However, it is possible that, in some situations, base-pairing interactions between sequences flanking individual ABS or FBS may further stabilize the conformational changes initiated by hnRNP proteins.

According to the looping-out model, the interaction between hnRNP A/B or between F/H proteins bound in the intron near splice junctions would represent a key step leading to efficient formation of a commitment complex. In vitro, the splicing efficiency of a small intron was not affected by ABS or by variations in the concentration of A1, possibly because the splice-site pairing step is not rate-limiting. Although the presence of ABS and FBS improved the splicing efficiency of enlarged introns in vitro, the activity of these elements may be more important in vivo when splicing decisions are taken co-transcriptionally. The hnRNP A/B or F/H–mediated looping out of intron sequences as they exit from the RNA polymerase II transcription complex may facilitate intron definition by improving the frequency of an encounter between a U1-bound 5′ splice site and a U2AF-bound 3′ splice site located downstream from the ABS–ABS or FBS–FBS complex.

The looping out of an intron may also occur in several steps, as would be expected when additional ABS or FBS are distributed along an intron. Given that a 5′ splice site located in between two ABS is repressed [7], the ABS- or FBS-mediated looping out of portions of introns may neutralize a multitude of weaker and/or non-productive interactions with pseudo or cryptic splice sites. However, if a splice site located between hnRNP-binding sites is strong enough, its commitment to an upstream 5′ splice site or to a downstream 3′ splice site may kinetically out-compete the looping-out process mediated by hnRNP proteins. The relative frequency of the two events would be expected to contribute towards setting alternative splicing profiles, and hence, may be influenced by the position of the splice sites relative to ABS or FBS, the speed of transcription, and the presence of silencers/enhancers flanking alternative splice sites.

Finally, the mechanism by which a looped-out splice site is repressed by flanking ABS or FBS remains unclear. The binding of U1 snRNP to a 5′ splice site is not greatly affected by flanking ABS or FBS. However, this mechanism of repression may be similar to the mechanism by which inverted repeats repress 5′ splice-site usage when substituted for ABS or FBS [7]. Commitment, or a later step of spliceosome assembly, may be compromised or delayed by a relatively rigid complex (ABS–ABS, FBS–FBS, or a duplex structure) whose topology may be incompatible with the structural flexibility necessary for efficient spliceosome assembly.

We are currently investigating whether heterotypic interactions can take place between hnRNP A/B and hnRNP F/H proteins as well as between other glycine-rich RNA-binding proteins. If so, such interactions may also play a role in remodeling the conformation of mammalian pre-mRNAs, with a significant impact on splicing efficiency and splice-site selection.

## Materials and Methods

### Oligonucleotides

The DNA primers used for the RT-PCR amplification of spliced products were 20 nt in length. E-Ad and BclX3 were used as downstream primers for the RT step and the PCR amplification of products carrying the adenovirus or Bcl-x as second exon, respectively. E-Ad (5′- GAGTTTGTCCTCAACCGCGA-3′) is complementary to the 5′ end of the adenovirus exon L2. BclX3 (5′- TCGGCTGCTGCATTGTTCCC-3′) is complementary to a region in Bcl-x exon 3. The upstream primer in all amplifications from in vitro splicing assays was a 21-nt oligonucleotide T3–5′ (5′- GGGAACAAAAGCTGGGTACCG-3′) that hybridizes near the 5′ end of all transcripts synthesized from the T3 RNA polymerase promoter.

Custom-made RNA oligonucleotides were purchased from Dharmacon Research (Lafayette, Colorado, United States). The 3′ half of the upstream oligonucleotide UA or UB is complementary to the intronic sequences at the 5′ end of the lambda insert A or B, respectively, 42 nt downstream from the 5′ splice site. These oligonucleotides have a CE1a element sequence at the 5′-end portion. Oligonucleotide UOA contains the same complementary sequences, but the CE1a element is located at the 3′ end. The downstream oligonucleotides Da and Db are complementary to a 20-nt region 67 nt upstream of the adenovirus exon L2, and 122 nt upstream of the Bcl-x exon 3, respectively. These oligonucleotides contain the CE1a element at their 3′-end portion. Oligonucleotide UBn shares its last 19 nt with oligonucleotide UB but has a non-ABS 25-nt tail at its 5′ end. Oligonucleotide UST has a 20-nt region at the 3′ end complementary to the intronic sequences between the distal and the proximal 5′ splice sites in RNA 553 [7], while the 5′ portion of this oligonucleotide contains the CE1a element.

2′*O*-Me oligonucleotides used in transfection assays include UV3A1, which is complementary to a 20-nt region starting 20 nt downstream from the 5′ splice site of exon V3. UV3A1 has the ABS from the CE1a element [43]. UV3NT contains the same complementary sequences as UV3A1 but lacks the ABS. UV3A1W, UV3A1W3, and UV3A1W5, respectively, contain two, three, and five consecutive ABS derived from the winner binding site for hnRNP A1 [20]. D16A1 and D16A1W, respectively, contain the CE1a and the winner A1-binding sites. mA1W5 carries five ABS but is complementary to the pre-mRNA of hnRNP A1. The sequences of all oligonucleotides used in splicing are shown in Table 2.

**Table 2:**
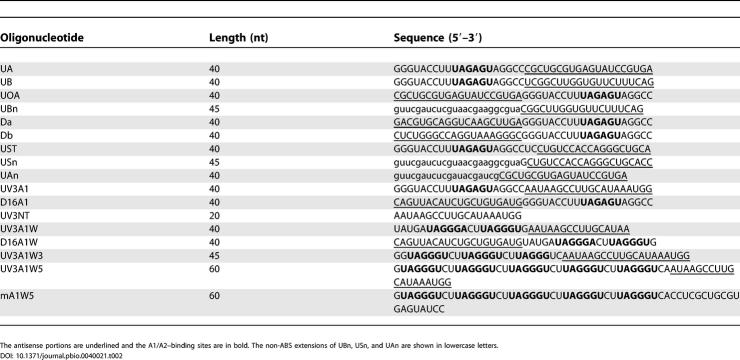
Sequence of the Antisense 2′*O*-Me RNA Oligonucleotides

### Transcription and splicing assays

Constructs containing the adenovirus exon L2 were linearized with ScaI, whereas constructs containing Bcl-x exon 3 were linearized using BglII and used as templates for in vitro transcription. In general, pre-mRNA substrates were synthesized in vitro using T3 RNA polymerase (USB) in the presence of minimal amounts of ^32^P-UTP and gel-purified as described previously [7]. A known amount of the pre-mRNA was then incubated in HeLa nuclear extract [44] under standard splicing conditions [24] at 30 °C. The RNA material was then extracted with phenol-chloroform-isoamylalcohol (PCA) and ethanol precipitated. To investigate the effect of RNA oligonucleotides on splicing, pre-mRNA molecules were mixed with either the individual oligonucleotide or with a mixture of the oligonucleotides prior to splicing. RNA species obtained after splicing were quantitated and resuspended in sterile water to a concentration of 5–10 atomoles per μl. An equivalent amount of this solution was then subjected to RT-PCR amplification. To analyze pre-mRNA splicing on conventional denaturing acrylamide gels, uniformly labeled pre-mRNAs were synthesized and processed as described previously [7].

The treatment of HeLa cells with siRNA to knockdown hnRNP F/H was performed as described previously [34]. Western blot analysis was performed using antibodies against hnRNP F and hnRNP H (kindly provided by Douglas Black). Recombinant His-tagged hnRNP F and hnRNP H proteins were produced from baculovirus-infected cells as described previously [34].

### RT-PCR

The pre-mRNAs incubated in splicing extracts were minimally labeled such that the amount of pre-mRNA used could be precisely quantitated and followed until after PCA extraction and ethanol precipitation. In many experiments, a small-intron pre-mRNA was co-incubated with the test pre-mRNA in splicing mixtures to assess equivalent recovery and loading. In some experiments, RNA controls were added only before the RT-PCR reaction. Amplification protocols used the ready-to-go RT-PCR beads (Amersham Pharmacia Biotech, Piscataway, New Jersey, United States) as described previously [7]. In several experiments, amplifications were performed in the presence of ^32^P-labeled dCTP. The reaction mixtures after amplification were treated with RNase A and the products were resolved on a 5% non-denaturing acrylamide gel, unless stated otherwise. The gel was stained with ethidium bromide and photographed under UV light. When amplified products were ^32^P-labeled, products were quantified on an InstantImager (Canberra-Packard, Meriden, Connecticut, United States) or a Storm PhosphorImager (Amersham Biosciences, Little Chalfont, United Kingdom) and then exposed on film.

### Immunoprecipitation assay

Plasmids p7-AdA(−.−) and (a.a) were linearized with BsmAI and transcribed with T3 RNA polymerase to generate uniformly labeled RNA. Briefly, splicing reactions containing 10^5^ counts per minute of ^32^P-labeled transcripts were incubated in a HeLa nuclear extract for 30 min at 30 °C, either in the presence or in the absence of oligonucleotides. Reactions were then placed on ice and incubated for 30 min with 1 μl of an antibody against hnRNP A1/A2 [45]. A quantity (50 μl) of protein A Sepharose (5 mg, Amersham Pharmacia Biotech) was added and the mixture was incubated for 15 min. After three washes with 1 ml of NET-2 buffer (50 mM Tris-HCl [pH 7.5], 150 mM NaCl, 0.5 mM DTT; and 0.05% Nonidet P-40), samples were PCA extracted and ethanol precipitated. The RNA recovered was fractionated on a denaturing 5% polyacrylamide gel.

### Gel shift and RNase H protection assays

The gel-shift assay with recombinant hnRNP proteins was carried out as described previously [43]. ΔU1 HeLa extracts were produced by addition of the 2′*O*-Me oligonucleotide CCU GCCAGGUAAGUA complementary to the 5′ end of U1 snRNA [46]. The oligonucleotide-mediated RNase H protection assay was conducted as described previously [24].

### Transfection and RNA analysis

COS-7 cells were cultured in Dulbecco's modified Eagle's medium, supplemented with 10% fetal bovine serum. Twenty-four hours prior to plasmid transfections, cells were seeded in six-well plates (35 mm) at a density of 2.5 × 10^5^ cells/well. At 50% confluence, the cells were transiently transfected with 2 μg of the purified plasmid p44:V3λλλλλλ [2] using Lipofectamine Plus (Invitrogen, Carlsbad, California, United States). Twenty-four hours later, the cells were treated with different 2′*O*-Me RNA oligonucleotides to a final concentration of 50 nM, and the cell culture was continued for 24 h.

Total RNA was prepared using TRIzol (Invitrogen) and treated with DNase I, according to the manufacturer's indications. Reverse transcription was performed with Omniscript RT (Qiagen, Valencia, California, United States) and the RT3 primer ( GAAGGCACAGTCGAGGCTG), which anneals to the 3′-UTR of the pcDNA3 vector to avoid the interference of the endogenous *CD44* mRNA. The reaction was carried out at 37 °C for 60 min, stopped at 95 °C for 5 min and ice-quenched, followed by PCR amplification in the presence of (α-^32^P)dCTP and of oligonucleotide primers directed to *CD44* exons 5 and 16 ( AGTGAAAGGAGCAGCACTTCAGG and TCAGATCCATGAGTGGTATGGGAC, respectively). The amplification procedure was as follows: 95 °C for 5 min, 35 cycles at 94 °C for 30 sec, 56 °C for 30 sec, and 72 °C for 30 sec; with a final extension at 72 °C for 15 min. Reaction products were resolved by electrophoresis in a non-denaturing 5% polyacrylamide gel and quantified using the InstantImager system (Canberra-Packard).

### Computational analysis

Human introns were from National Center for Biotechnology Information (NCBI) human genome build 35.1 (http://www.ncbi.nlm.nih.gov). A total of 156,525 introns of sizes greater than 330 bp were retained for further analysis. One hundred rounds of shuffling were carried out in selected portions (+11 to +150 and −41 to −180) of all introns to calculate random occurrence and standard deviations.

## Supporting Information

Figure S1 RT-PCR Assay of Splicing Mixtures Incubated with Different Ratios of Test and Control Pre-mRNAs A quantity (80 pM) of test 7-AdA(−.−) or 7-AdA (+.+) pre-mRNA was mixed with various amounts of control 7B-Ad pre-mRNA (from 0 to 40 pM). The mixtures were incubated in HeLa nuclear extracts for 2 h at 30 °C. The RT-PCR assay was carried out with a single pair of primers that amplify mRNA products derived from all pre-mRNAs. The stimulation provided by ABS can be observed at all ratios of test and control pre-mRNAs.
(201 KB EPS).

Figure S2 Gel-Shift Assay Using Recombinant hnRNP H, F, and A1 The initial G1 and G2 elements (larger letters) stimulated splicing when inserted into a *Fugu* intron. The sequences immediately flanking the insertion sites are also shown. The 16-nt G1 and 19-nt G2 RNA oligonucleotides were 5′-end labeled and individually incubated with hnRNP proteins (1, 2, and 3 μM) in the presence of heparin. Complexes were fractionated on a native 5% acrylamide gel.
(431 KB EPS).

## Abbreviations

ABS: hnRNP A/B–binding site
CIHR: Canadian Institutes of Health Research
FBS: hnRNP F/H–binding site
nt: nucleotide
PCA: phenol-chloroform-isoamylalcohol
RRM: RNA-recognition motif
SR: serine/arginine–residue proteins
